# Randomized Phase II Study of Duligotuzumab (MEHD7945A) vs. Cetuximab in Squamous Cell Carcinoma of the Head and Neck (MEHGAN Study)

**DOI:** 10.3389/fonc.2016.00232

**Published:** 2016-10-31

**Authors:** Jérôme Fayette, Lori Wirth, Cristina Oprean, Anghel Udrea, Antonio Jimeno, Danny Rischin, Christopher Nutting, Paul M. Harari, Tibor Csoszi, Dana Cernea, Paul O’Brien, William D. Hanley, Amy V. Kapp, Maria Anderson, Elicia Penuel, Bruce McCall, Andrea Pirzkall, Jan B. Vermorken

**Affiliations:** ^1^Centre Léon Bérard, Université de Lyon, Lyon, France; ^2^Massachusetts General Hospital, Boston, MA, USA; ^3^Oncomed SRL, Timisoara, Romania; ^4^Medisprof SRL, Cluj-Napoca, Romania; ^5^University of Colorado Cancer Center, Aurora, CO, USA; ^6^Peter MacCallum Cancer Centre, University of Melbourne, Melbourne, VIC, Australia; ^7^Royal Marsden NHS Trust, The Institute of Cancer Research London, Sutton, UK; ^8^University of Wisconsin Hospital and Clinics, Madison, WI, USA; ^9^Jász-Nagykun-Szolnok Megyei Hetényi Géza Kórház, Szolnok, Hungary; ^10^Institutul Oncologic Prof. Dr. Ion Chiricuta Cluj-Napoca, Cluj-Napoca, Romania; ^11^Medical University of South Carolina, Charleston, SC, USA; ^12^Genentech, South San Francisco, CA, USA; ^13^Antwerp University Hospital, Edegem, Belgium

**Keywords:** NRG1, HER3, EGFR, SCCHN, HPV, duligotuzumab, MEHD7945A, cetuximab

## Abstract

**Background:**

Duligotuzumab, a novel dual-action humanized IgG1 antibody that blocks ligand binding to epidermal growth factor receptor (EGFR) and human epidermal growth factor receptor 3 (HER3), inhibits signaling from all ligand-dependent HER dimers, and can elicit antibody-dependent cell-mediated cytotoxicity. High tumor-expression of neuregulin 1 (NRG1), a ligand to HER3, may enhance sensitivity to duligotuzumab.

**Methods:**

This multicenter, open-label, randomized phase II study (MEHGAN) evaluated drug efficacy in patients with recurrent/metastatic (R/M) squamous cell carcinoma of the head and neck (SCCHN) progressive on/after chemotherapy and among patients with NRG1-high tumors. Patients received duligotuzumab (1100 mg IV, q2w) or cetuximab (400 mg/m^2^ load, 250 mg/m^2^ IV, q1w) until progression or intolerable toxicity. Tumor samples were assayed for biomarkers [*NRG1, ERBB3*, and human papillomavirus (HPV) status].

**Results:**

Patients (*N* = 121) were randomized (duligotuzumab:cetuximab; 59:62), median age 62 years; ECOG 0–2. Both arms (duligotuzumab vs. cetuximab, respectively) showed comparable progression-free survival [4.2 vs. 4.0 months; HR: 1.23 (90% confidence interval (CI): 0.89–1.70)], overall survival [7.2 vs. 8.7 months; HR 1.15 (90% CI: 0.81–1.63)], and objective response rate (12 vs. 14.5%), with no difference between patients with *NRG1*-high tumors or *ERBB3*-low tumors. Responses in both arms were confined to HPV-negative patients. Grade ≥3 adverse events (AEs) (duligotuzumab vs. cetuximab, respectively) included infections (22 vs. 11.5%) and GI disorders (17 vs. 7%), contributing to higher rates of serious AEs (41 vs. 29.5%). Metabolic disorders were less frequent with duligotuzumab (10 vs. 16%); any grade rash-related events were less with duligotuzumab (49 vs. 67%).

**Conclusion:**

While several lines of preclinical evidence had supported the premise that the blockade of HER3 in addition to that of EGFR may improve outcomes for patients with R/M SCCHN overall or specifically in those patients whose tumors express high levels of *NRG1*, this study provided definitive clinical evidence refuting this hypothesis. Duligotuzumab did not improve patient outcomes in comparison to cetuximab despite frequent expression of *NRG1*. These data indicate that inhibition of EGFR alone is sufficient to block EGFR–HER3 signaling, suggesting that HER2 plays a minimal role in this disease. Extensive biomarker analyses further show that HPV-negative SCCHN but not HPV-positive SCCHN are most likely to respond to EGFR blockage by cetuximab or duligotuzumab.

## Introduction

Multiple members of the HER family receptor tyrosine kinases, including epidermal growth factor receptor (EGFR), HER1, and HER2, are established therapeutic targets in several epithelial malignancies (1). EGFR is a rational focus for squamous cell carcinoma of the head and neck (SCCHN) given the prevalence of its overexpression and crucial role in SCCHN pathogenesis (2, 3). Moreover, EGFR is a clinically validated therapeutic target in recurrent/metastatic SCCHN with approval of cetuximab as a single agent (4), concurrent with platinum-based chemotherapy (5), and in combination with radiotherapy in the curative SCCHN setting (6). Many SCCHN patients do not respond to cetuximab therapy, and for those who do, they commonly manifest acquired resistance following prolonged exposure to the drug. These findings have prompted the design of next-generation EGFR inhibitors and approaches that may serve to overcome resistance to cetuximab.

Factors thought to contribute to cetuximab resistance in SCCHN patients include upregulation of ligands for EGFR and human epidermal growth factor receptor 3 (HER3) (7, 8), heterodimerization of EGFR and HER2 with HER3 (9), overexpression of HER2 and HER3 (10), and overexpression and aberrant nuclear localization of EGFR (11). Interestingly, a subset of SCCHN cell lines are resistant to anti-EGFR tyrosine kinase inhibitor (TKI) treatment and do not overexpress HER2 but are sensitive to combined anti-EGFR/anti-HER2 TKI inhibition (12). Many of these cell lines were found to have high expression of heregulin (HRG), the ligand binding to HER3, and activation of HER3 signaling. It was hypothesized that such cells may escape the effects of anti-EGFR therapy *via* HRG-dependent signaling of a HER2/HER3 dimer. An analysis of >700 tumor samples from patients with non-small cell lung cancer (NSCLC), SCCHN, colorectal, breast, or ovarian cancer found that median HRG mRNA expression is significantly higher in SCCHN tumors than in the other tumor types (13). HRG represents alpha and beta forms of neuregulin 1 (14) and is here forth referred to as NRG1.

Duligotuzumab (MEHD7945A) is a novel dual-action human IgG1 monoclonal antibody that simultaneously targets HER3 and EGFR (15). HER3 is encoded by the *ERBB3* gene. Duligotuzumab demonstrated superior activity compared with mono-specific EGFR- or HER3-targeting antibodies in the non-clinical FaDu SCCHN model (16), as well as in human xenograft models derived from SCCHN and NSCLC tumors with acquired resistance to EGFR inhibitors (17). Preliminary evidence of clinical activity included two confirmed partial responses (PRs) in SCCHN patients (18) who had entered the duligotuzumab phase Ia study after progressing on prior therapy, one having relapsed after multiple prior treatment regimens including an EGFR inhibitor. Both patients’ tumors were found to have *NRG1* expression near the top of the range observed in the analysis of tumor samples described above.

Taken together, these observations suggested that the addition of HER3 blockade to EGFR blockade with duligotuzumab may improve clinical outcomes in patients with recurrent or metastatic (R/M) SCCHN overall or specifically in those patients whose tumors express high levels of NRG1. This phase II study evaluated the efficacy of duligotuzumab vs. cetuximab in patients with R/M SCCHN progressive on/after chemotherapy and included *post hoc* analyses by *NRG1* expression levels, *ERBB3* expression levels, and human papillomavirus (HPV) status.

## Methods

### Patients

Eligible patients were ≥18 years of age with histologically confirmed R/M SCCHN who had progressed after one or more lines of treatment, at least one platinum-based regimen for R/M disease, and not suitable for local therapy. Patients with ECOG performance status of 0, 1, or 2, disease measurable per RECIST v1.1, adequate hematologic, renal, or hepatic function, no prior HER targeted therapy with exception of EGFR inhibitor given in upfront setting and as long as discontinued ≥3 months prior to enrollment were included. Patients were excluded if they had nasopharyngeal cancer.

### Study Design

**Figure 1:**
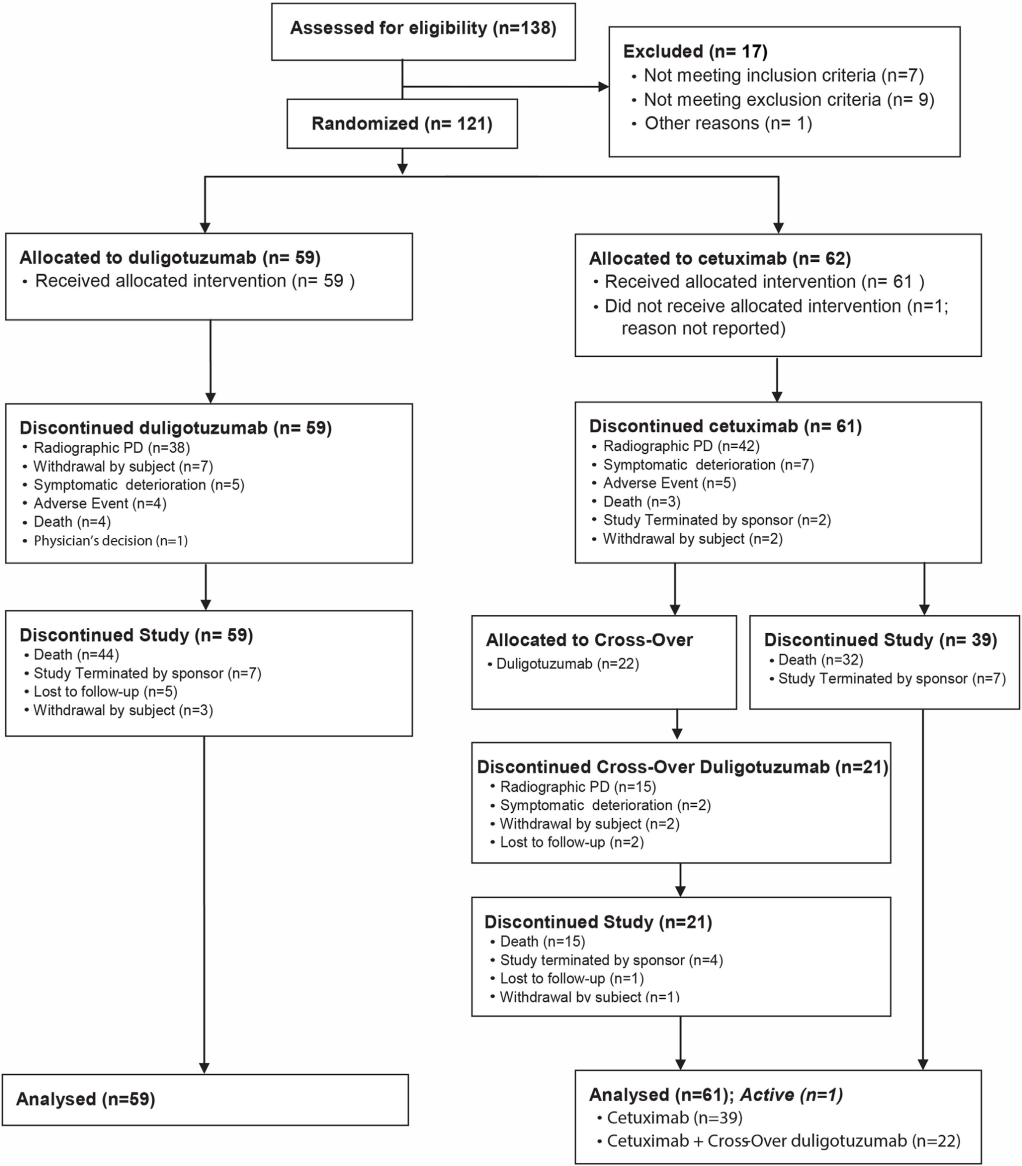
**Study design**.

This was a phase II, randomized, multicenter, open-label study with two arms (Figure 1) assessing duligotuzumab vs. cetuximab in R/M SCCHN patients. Institutional review boards at all participating institutions approved the study protocol. All patients gave written informed consent. The study was conducted according to good clinical practice (GCP), and the Declaration of Helsinki and its amendments, and was registered at http://ClinicalTrials.gov, number NCT01577173 (19).

Patients received duligotuzumab, 1100 mg IV, administered every 2 weeks (Arm A), or cetuximab, 400 mg/m^2^ loading dose, 250 mg/m^2^ IV, administered weekly (q1w) (Arm B). Patients were randomized to one of the two treatment arms in a 1:1 ratio using an interactive voice response system (IVRS). Stratification factors included ECOG 0/1 vs. 2 and time to platinum failure (≤2 vs. >2 months). Patients were treated with either study drug until disease progression or other unacceptable toxicity. Patients with disease progression on Arm B (cetuximab) could cross over to Arm A (duligotuzumab) upon central confirmation of progressive disease (PD) (RECIST v1.1), and as long as principal eligibility criteria were met.

The primary endpoint was progression-free survival (PFS) by investigator assessment in all randomized patients [intention to treat (ITT) population] and in the subset with highest *NRG1* expression in the tumor. *NRG1* was assessed by qRT-PCR at Genentech. Secondary objectives included overall survival (OS), overall response rate (ORR), safety/tolerability, and characterization of pharmacokinetics (PKs) and anti-therapeutic antibodies (ATA) from patients treated on Arm A. Exploratory objectives included assessing tumor samples from patients for the prevalence and potential prognostic significance of *NRG1* and *ERBB3*, and HPV status and its potential association with antitumor activity.

### Safety

Safety was assessed by incidence, nature, severity, and relatedness of adverse events (AEs) and graded for severity (NCI-CTCAE v4.0). All patients who received ≥1 dose of study treatment were included in the safety evaluation. The following events were categorized as of special interest: grade ≥3 events associated with infusion-related reactions (defined as AEs within 24 h of infusion and attributed to study drug), grade ≥3 rash, and grade ≥3 diarrhea.

### Pharmacokinetics

Serum samples were collected at screening, day 1 of cycles 1–8, and treatment completion. Duligotuzumab concentration was determined using a qualified enzyme-linked immunosorbent assay with the minimum quantifiable concentration of 150 ng/mL. PK parameters were derived from non-compartmental analysis (NCA) (WinNonlin version 5.2.1.). A population PK analysis of duligotuzumab from phase Ia study DAF4873g (data on file) demonstrated that body weight has only a minor impact on PK parameters [volume of distribution (Vd) and clearance (CL)], thus supporting that flat dosing would have little effect on duligotuzumab PK variability in comparison with weight-based dosing.

Serum anti-duligotuzumab antibody (ATA) samples were collected prior to duligotuzumab infusion on day 1 of cycles 1–8, and at the study termination visit, and were analyzed using a validated bridging antibody immunoassay that could detect surrogate anti-duligotuzumab positive-control antibody (249 ng/mL) in the absence of duligotuzumab.

### Biomarker Analyses

Mandatory archival (or fresh as available) tumor tissues were evaluated to characterize the disease biology and to identify potential predictive biomarkers for improved outcomes with duligotuzumab compared to cetuximab, with particular attention to the HER3 ligand, NRG1. For *NRG1* and receptor tyrosine kinase erbB-3 (*ERBB3*), RNA expressions were measured by both qRT-PCR and by dual-colored *in situ* hybridization (ISH). Additionally, HPV detection was performed by qRT-PCR.

### Efficacy

Tumor assessments were performed at screening and during every 6 weeks of study treatment (e.g., prior to cycle 4 and every 6 weeks thereafter), and at the treatment completion visit for patients who discontinue for reason other than PD. Response assessments were performed by the investigators according to RECIST v1.1. The primary endpoint was PFS, defined as the time from randomization to the first occurrence of progression or death, whichever occurred first. Secondary endpoints included OS (defined as the time from randomization to death) and objective response defined as complete response (CR) or PR confirmed ≥4 weeks after initial documentation. Unless stated otherwise, analyses excluded data after crossover.

### Sample Size

This trial was designed to obtain informative estimates of the PFS hazard ratios in the overall patient population, and the NRG1-high patient population to enable further decision making, without adequate power to detect the minimum clinically meaningful difference between the treatment arms at a statistically significant type 1 error level (α = 0.05). Therefore, the utility of formal hypothesis testing was limited because statistically negative outcomes did not necessarily exclude clinically significant treatment effects. Consequently, 90% CIs for the PFS hazard ratios were calculated. In order to observe 90 disease progression or death events in all patients and 35 disease progression or death events in the NRG1-high patients, the study was planned to enroll 110 patients, assuming the prevalence of NRG1-high patients was 40%. For a lower prevalence, additional patients could be enrolled to meet the required number of disease progression or death events in the HRG-high patient population.

### Statistical Analysis

Safety analyses included all patients who received any amount of study treatment. Efficacy analyses were conducted on all randomized patients and all randomized NRG1-high patients. For each time-to-event endpoint, Kaplan–Meier analysis was used to estimate the medians in each treatment arm, and Cox regression was used to estimate the hazard ratio.

## Results

### Patient Population

From July 13, 2012 to July 09, 2013, 121 patients were randomized (59 Arm A, 62 Arm B), of whom 120 were evaluable for safety, 108 for biomarker analysis, and 22 during the crossover phase. The cutoff date for analysis was March 02, 2015, resulting in a minimum follow-up of 19.8 months.

Key baseline characteristics included the stratification factors, ECOG, and time to PD, since most recent platinum treatment were well-balanced overall (Table 1) with the exception of fewer HPV-positive patients and fewer patients with locoregional recurrence only who enrolled on the experimental duligotuzumab arm, as compared to the cetuximab arm. Upon review of staging and prior medical history, it was identified that 25/121 patients were enrolled in the first line (1L) rather than the protocol specified 2L + R/M setting. Those patients had not received prior platinum-based therapy for *de novo* metastatic (Stage IVc) or for recurrence of locally advanced disease, but rather in the adjuvant or definitive setting. Subset analyses excluding those 25 patients showed no significant impact on the efficacy results.

**Table 1:** **Patient characteristics**.

	Duligotuzumab (*n* = 59)	Cetuximab (*n* = 62)	All patients (*N* = 121)
**Age (years)**			
Median (range)	62.0 (29–80)	62.0 (28–84)	62.0 (28–84)
**Sex**			
Male	55 (93%)	46 (75%)	101 (84%)
**Race**			
White	47 (80%)	45 (74%)	92 (77%)
**Tobacco use history**			
Never	12 (20%)	8 (13%)	20 (17%)
**ECOG performance status**^a^			
0/1	50 (85%)	52 (85%)	102 (85%)
**Time to progressive disease since last platinum-based chemo**^a^			
≤2 months	31 (53%)	34 (55%)	65 (54%)
**Site of primary tumor**			
Oral cavity	15 (25%)	20 (32%)	35 (29%)
Oropharynx	16 (27%)	20 (32%)	36 (30%)
Larynx	11 (19%)	8 (13%)	19 (16%)
Hypopharynx	6 (10%)	6 (10%)	12 (10%)
Head and neck	7 (12%)	5 (8%)	12 (10%)
Unknown primary site	4 (7%)	3 (5%)	7 (6%)
**HPV (qRT-PCR assay)**			
Positive	10 (17%)	15 (24%)	25 (21%)
**Prior therapies, *n* (%)**			
Radiation therapy	52 (88%)	52 (84%)	104 (86%)
**Number of prior systemic regimen**			
Median (range)	2 (1–5)	1 (1–5)	1 (1–5)
**Extent of disease at baseline**			
Locoregional recurrence only	7 (12%)	14 (23%)	21 (17%)

*^a^Stratification factor*.

### Study Treatment

The median number of doses given of duligotuzumab was 7 (range 1–24) and of cetuximab 12 (range 1–59). The median treatment duration for duligotuzumab and cetuximab was 12.4 and 12.0 weeks, respectively. The median relative cumulative dose intensity for duligotuzumab and cetuximab was 87.5 and 89.8%, respectively. Treatment modification or interruption was similar for duligotuzumab and cetuximab, with 42 (71%) patients missing at least one dose in the duligotuzumab arm and 49 (80%) in the cetuximab arm. There were 2 (3%) dose modifications in the duligotuzumab arm and 11 (18%) in the cetuximab arm.

The majority of study treatment discontinuations were due to radiographic disease progression or symptomatic deterioration [73% duligotuzumab (radiographic PD: 64%); 80% cetuximab (radiographic PD: 69%)]. Twenty-two patients crossed over to duligotuzumab after central confirmation of progression on cetuximab. Median treatment duration and cumulative dose intensity were 7.6 months and 88.9% for duligotuzumab, respectively. One patient continues duligotuzumab in the crossover arm.

### Safety

The nature and incidence of AEs in the 120 patients evaluable for safety regardless of attribution are summarized (Table 2). The most common AEs were rash (and related terms), infections (MedDRA System Order Class), diarrhea, fatigue, and nausea. The safety profile of duligotuzumab was largely similar to that of cetuximab, however, relative to cetuximab, duligotuzumab was associated with less rash (49 vs. 65%), but more diarrhea (42 vs. 25%), mucosal inflammation (22 vs. 8%), and infection (59 vs. 49%). Grade ≥3 AEs were more frequent in the duligotuzumab arm (61%) vs. the cetuximab arm (51%). Seven deaths (five in the duligotuzumab arm; two in the cetuximab arm), none of which were assessed as related to study treatment, were attributed to underlying disease, disease progression, or an unknown cause.

**Table 2:** **Adverse events reported in ≥15% of patients in either arm regardless of attribution**.

	Duligotuzumab (*n* = 59)		Cetuximab (*n* = 61)	
MedDRA preferred term	Any grade	Grade ≥3	Any grade	Grade ≥3
All	58 (98%)	36 (61%)	59 (97%)	31 (51%)
Rash and related MedDRA Terms^a^	29 (49%)	–	40 (66%)	4 (7%)
Infections and infestations MedDRA SOC^b^	35 (59%)	13 (22%)	30 (49%)	7 (12%)
Diarrhea	25 (42%)	2 (3%)	15 (25%)	–
Fatigue	19 (32%)	2 (3%)	18 (29.5%)	1 (2%)
Nausea	13 (22%)	–	18 (29.5%)	–
Hypomagnesemia	12 (20%)	1 (2%)	16 (26%)	3 (5%)
Vomiting	12 (20%)	–	11 (18%)	–
Pyrexia	15 (25%)	–	6 (10%)	–
Headache	15 (25%)	–	6 (10%)	–
Skin fissures	11 (19%)	–	8 (13%)	1 (2%)
Decreased appetite	10 (17%)	1 (2%)	10 (16%)	1 (2%)
Mucosal inflammation	13 (22%)	–	5 (8%)	–
Weight decreased	6 (10%)	–	10 (16%)	1 (2%)
Constipation	9 (15%)	–	7 (11.5%)	–
Dry skin	8 (14%)	–	10 (16%)	–
Dyspnea	7 (12%)	1 (2%)	11 (18%)	4 (7%)
Asthenia	6 (10%)	1 (2%)	10 (16%)	1 (2%)

*AEs presented are prior to crossover; AEs in crossover arm not reported here but similar*.

*^a^Rash and related MedDRA terms = dermatitis acneiform, rash, rash maculo-papular, rash macular, rash erythematous, rash pruritic, rash generalized, rash papular, rash pustular, genital rash, and mucocutaneous rash*.

*^b^MedDRA System Order Class Infections and Infestations terms: paronychia (22% in duligotuzumab arm vs. 10% in cetuximab arm; No Grade 3 AEs), conjunctivitis, pneumonia, respiratory tract infection, bronchitis, lower respiratory tract infection, oral candidiasis, *Candida* infection, folliculitis, oral fungal infection, upper respiratory tract infection, cellulitis, sepsis, urinary tract infection, device-related infection, infection, nasopharyngitis, lung infection, nail infection, pharyngitis, oral herpes, abscess, abscess neck, bacteremia, *Clostridium difficile* infection, ear infection, fungal infection, groin abscess, infected bites, influenza, meningitis, mucosal infection, onychomycosis, osteomyelitis, rash pustular, rhinitis, skin bacterial infection, skin infection, staphylococcal infection, viral upper respiratory tract infection, abscess limb, acute sinusitis, bacteriuria, furuncle, herpes zoster, infected fistula, oral infection, pneumonia necrotizing, sinusitis, stoma site infection, and tracheostomy infection*.

Infusion-related reactions of headache and fever (events observed within 24 h of treatment and reported as related to study treatment; grade 1–2) had a higher incidence in the duligotuzumab arm (13 vs. 3% for headache and 10 vs. 5% for fever). No allergic type infusion-related reactions were observed in the duligotuzumab arm; anaphylactic reactions to cetuximab were reported in 3% of patients. Discontinuations due to AEs were higher in the cetuximab arm (7% duligotuzumab and 13.5% cetuximab).

### Pharmacokinetics

Target exposures of duligotuzumab were achieved in 100% of patients. The mean steady state (cycle 4) duligotuzumab PK parameter values (SD) for predose and C_max_ were 82.4 (40.4) and 366 (106) μg/mL, respectively. There was a weak trend observed in PK vs. body weight. No PK differences were detected in patient subpopulations (*NRG1* high/low or HPV+/−). ATAs were detected in 2/61 patients (3.3%) and had no impact on PK or safety.

### Clinical Activity

**Figure 2:**
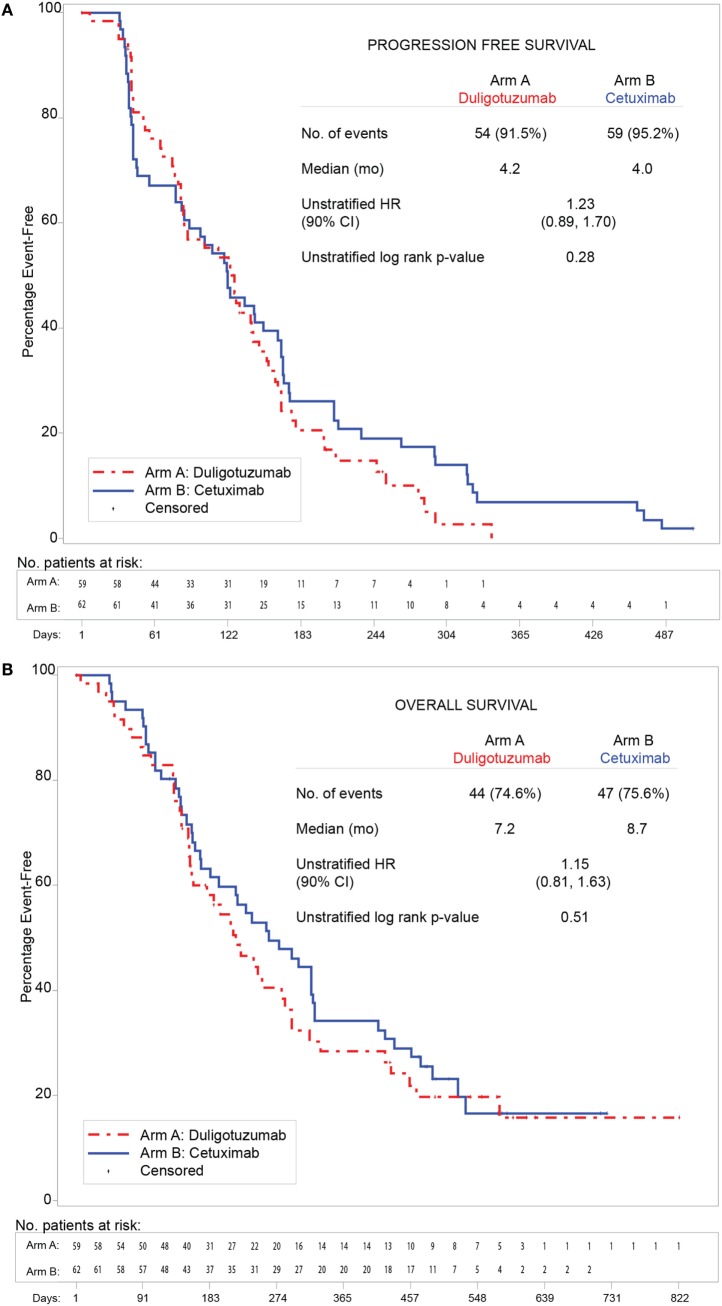
**Duligotuzumab vs. cetuximab in intention to treat (ITT) population showing comparable antitumor activity**. **(A)** Progression-free survival. **(B)** Overall survival.

**Figure 3:**
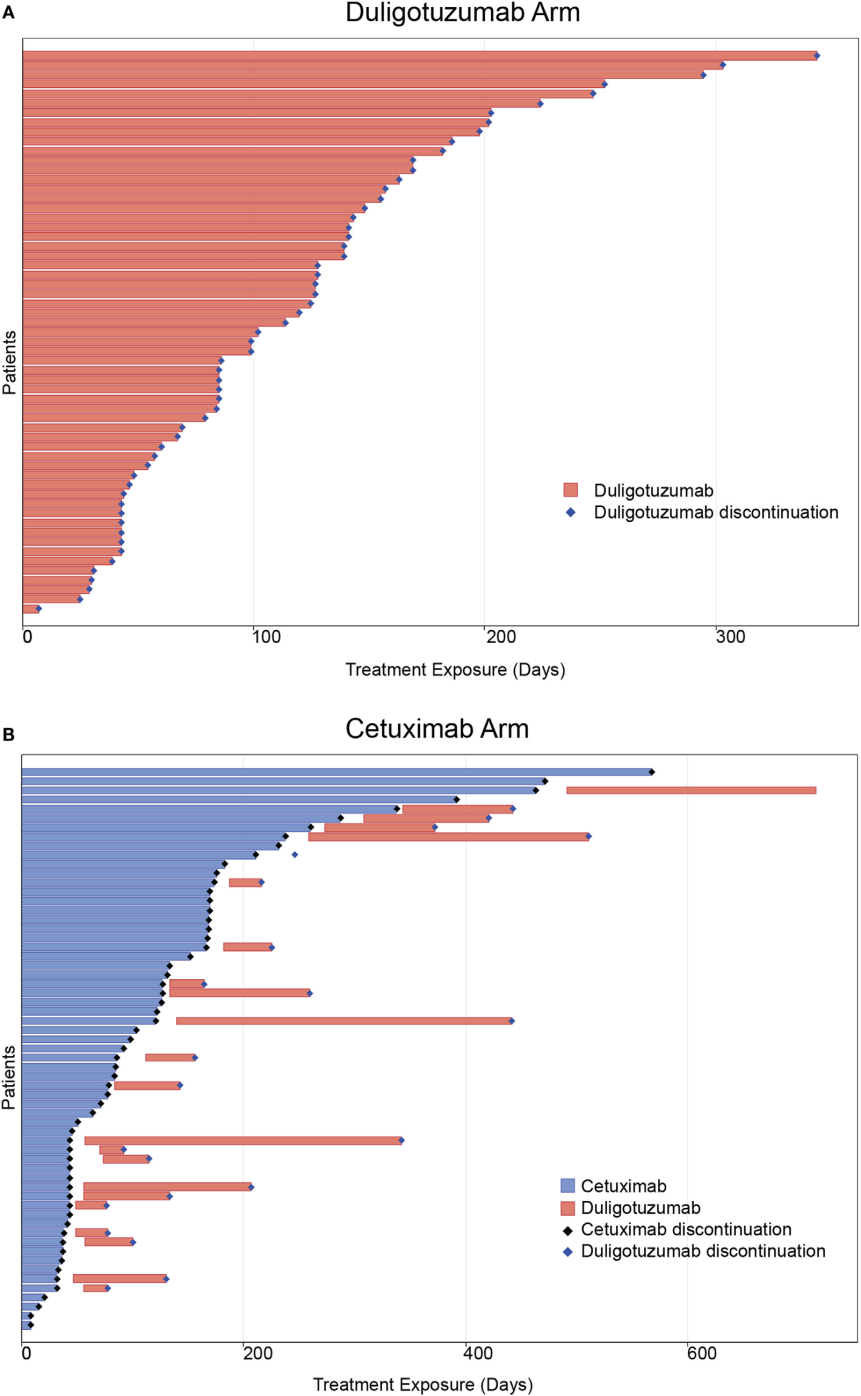
**ORR and treatment duration for primary treatment arms and crossover patients**. **(A)** Duligotuzumab arm. **(B)** Cetuximab arm.

In the ITT population, duligotuzumab and cetuximab showed comparable PFS [median 4.2 vs. 4.0 months, respectively, unstratified HR 1.23 (90% CI 0.89–1.70)] and OS [median 7.2 vs. 8.7 months, respectively, unstratified HR 1.15 (90% CI 0.81–1.63)] (Figure 2). PFS and OS subgroup analysis by patient and disease characteristics and stratification factors (ECOG and time to PD since most recent platinum treatment) did not show benefit for duligotuzumab over cetuximab. Objective response and treatment duration were comparable: ORR 12% (7 patients) duligotuzumab vs. 14.5% (9 patients) cetuximab, duration of objective response was median 5.4 months (duligotuzumab) vs. 4.3 months (cetuximab). There was limited evidence of activity in crossover patients (*n* = 22) (Figure 3).

Best responses in the duligotuzumab arm included 1 (2%) CR, 8 (14%) PRs, and 36 (61%) individuals with stable disease. The cetuximab arm included 2 (3%) CRs, 11 (18%) PRs, and 25 (40%) individuals with stable disease. Of 22 patients who crossed over to duligotuzumab after cetuximab failure, 10 showed stable disease (45%), exceeding prior PFS on cetuximab in 5 patients, and with one patient remaining active on study.

### Biomarker Subset Analyses

**Figure 4:**
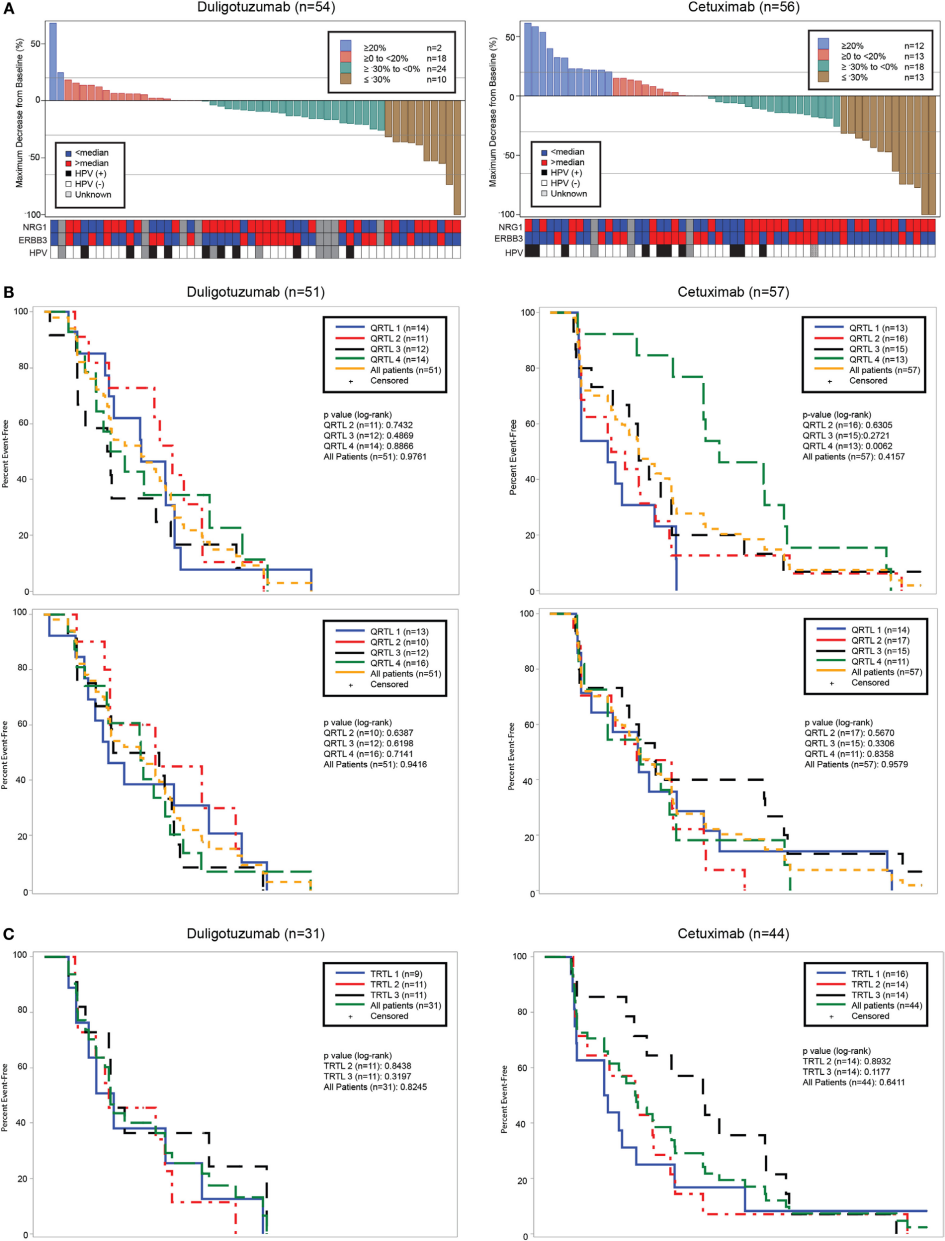
**Efficacy relative to key biomarkers: (A) best change in target lesion sum of longest dimension (SLD) and corresponding *NRG1, ERBB3*, and HPV status; (B) PFS by *NRG1* and *ERBB3* expression levels by quartiles; and (C) *NRG1* measured by qRT-PCR or by ISH does not predict response to duligotuzumab**.

Biomarker subtype analysis was performed for *NRG1* and *ERBB3* in 108 tumor samples (89%; 51/59 in the duligotuzumab arm and 57/62 in the cetuximab arm), both potential indicators of HER3 activity. Baseline expression using median and quartile cutoffs, respectively, was assessed relative to best change in sum of longest diameters of lesions from baseline and PFS. While there appeared to be a trend toward elevated *NRG1* levels and an increase in tumor shrinkage (Figure 4A), neither elevated *NRG1* nor reduced *ERBB3* expression predicted for response to duligotuzumab or against response to cetuximab (Figure 4B). Moreover, the trend toward elevated *NRG1* expression and increased tumor shrinkage was observed in both study arms. Similar observations were made for OS. Of note, *NRG1* expression levels were comparable to those in the phase Ia study (including responders) (18). To further address the hypothesized and preclinically supported role for *NRG1* autocrine expression in SCCHN, dual-colored ISH (*NRG1* and *ERBB3*) was performed and the biomarker analysis was repeated. Similar to expression analysis, co-localized expression of *NRG1* and *ERBB3* did not predict for response to duligotuzumab (Figure 4C).

**Figure 5:**
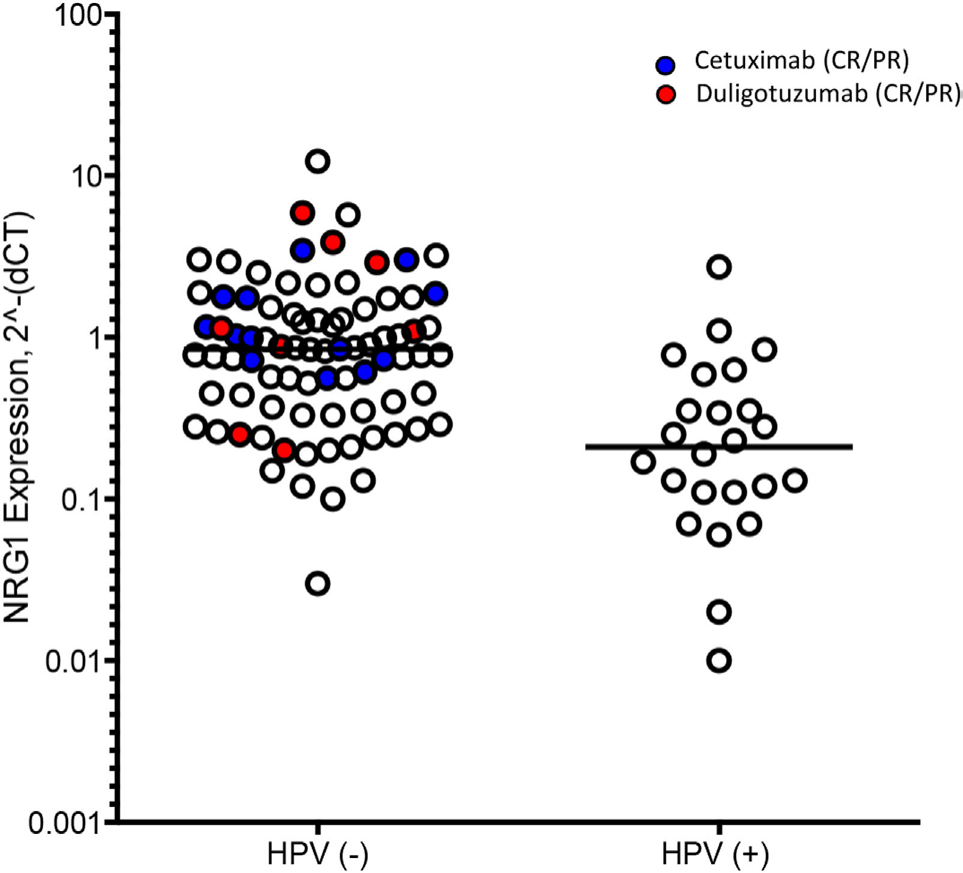
**HER family ligand expression by HPV status**. Ligand expression was higher in HPV-negative (−) subjects, with no responses in HPV-positive (+).

In addition to molecular biomarkers, HPV status based on qRT-PCR methodology was performed. Of 110 evaluable tumor samples from both treatment arms, 25 were HPV-positive and 85 HPV-negative. HPV-negative samples tended to have higher NRG1 ligand expression. Of the 25 patients with HPV-positive tumors, the site of primary tumor was oropharynx (14), oral cavity (4), larynx (1), and other or unknown locations (6). Among HPV-positive patients treated with either duligotuzumab or cetuximab, there were no responses (Figure 5).

## Discussion

The results of this phase II study show that dual inhibition of HER3 and EGFR by single-agent duligotuzumab demonstrated activity comparable, but not superior, to single-agent cetuximab in second or more line R/M SCCHN. High *NRG1* gene expression in tumor (primary biomarker hypothesis) was not associated with duligotuzumab efficacy as response rates, and PFS was similar between groups. Similarly, no association was found with *ERBB3* gene expression. Target study drug exposures were achieved for duligotuzumab. The phase II fixed dose of 1100 mg q2w duligotuzumab, which provides equivalent exposure to the phase Ia expansion dose of 14 mg/kg q2w, was selected based on achieving PK targets in >80% of patients. PK targets included an AUC based on xenograft models (equivalent to 300 day × μg/ml) and a C-trough of 5.3 μg/ml, which is predicted to correspond to 90% target saturation and 95% receptor binding. There was no clear relationship between exposure to duligotuzumab and safety or efficacy.

The safety profile of duligotuzumab was largely similar to cetuximab, with few exceptions. While duligotuzumab was associated with fewer rash events (49 vs. 65%) compared to cetuximab, GI disorders were more frequent with duligotuzumab vs. cetuximab (diarrhea 42 vs. 25%; mucosal inflammation 22 vs. 8%). These observations are consistent with other regimens targeting multiple HER receptors, and likely indicative of HER3-mediated toxicity [e.g., pan-HER TKI afatinib vs. cetuximab in R/M SCCHN (20); addition of pertuzumab (inhibitor of the dimerization of HER2/HER3) to trastuzumab and chemotherapy in 1L HER2 + breast cancer (21)]. MedDRA System Order Class “Infections and Infestations” were more frequent in the duligotuzumab arm (all grades: 59 vs. 49%; grades ≥3: 22 vs. 12%) with no particular pattern of infection identified in either treatment arm. The higher frequency in duligotuzumab-treated patients may be related to this groups’ higher rate of mucosal inflammation.

Despite strong preclinical data coupled with activity in phase I suggesting a potential role of HER3 signaling in SCCHN (16, 18), the dual inhibition of EGFR and HER3 was not associated with improved efficacy in this study, neither in all randomized nor biomarker positive patients. The outcome was similar in study of the pan-HER TKI, afatinib vs. cetuximab in all-comer ≥1L SCCHN (20), with median PFS 13.0 vs. 15.0 weeks (HR 0.93, 95% CI 0.62–1.38, *p* = 0.71). Median PFS for patients who crossed over to afatinib was 9.3 and 5.7 weeks for those crossing to cetuximab (HR 0.64, 95% CI 0.38–1.05, *p* = 0.08). Taken together, these data suggest that the inhibition of HER3 in addition to EGFR does not significantly improve response in recurrent/metastatic SCCHN. Alternatively, these data may indicate that inhibition of EGFR alone may be sufficient to block EGFR–HER3 signaling, suggesting that HER2 plays a minimal role in this disease. A better understanding of receptor dimerization following exposure to different agents may provide critical insights to this mechanism. It is also possible that HER3 signaling has a moderate role in SCCHN and inhibition of HER3 by duligotuzumab compensated for its slightly lower affinity for EGFR (*K*_d_ of 0.4 nM vs. 0.39 nM for cetuximab) (16, 22). Finally, there is no evidence for differential ADCC activity as duligotuzumab demonstrated mediated ADCC activity in cell lines with high EGFR expression that was similar to what has been described with cetuximab (16).

The potential for enhanced chemosensitization and radiosensitization with dual HER3/EGFR inhibition remains an unanswered question. Preclinical studies showed enhanced radiation response with dual HER3/EGFR inhibition in SCCHN and lung cancer model systems (23). Chemotherapy-induced upregulation of NRG1 and activation of HER3 have been reported *in vitro* (24). A phase Ib study combining duligotuzumab with either cisplatin/5FU or carboplatin/paclitaxel in 1L R/M, SCCHN showed encouraging activity with ORR of 67% though without clear relationship with evaluated biomarkers and hence no means for patient selection (25, 26). While the phase Ib study showed high antitumor efficacy and a higher frequency and severity of select AEs in comparison to historical phase III data on combination of chemotherapy and EGFR inhibitors, it is not clear if such was owed to chemo sensitization or rather was reflective of the small sample size.

The role of EGFR inhibition in HPV-associated oropharyngeal cancer vs. HPV-negative head and neck cancer remains controversial and may differ depending on whether given as monotherapy or combined with chemotherapy or radiotherapy. Our findings of no objective responses in the HPV-positive group with either agent are consistent with those recently reported with afatanib (27), with no objective responses in the p16-positive population and a 13.5% response rate in the p16-negative population, and raise further doubts about the role of anti-EGFR monotherapy for R/M HPV-associated oropharyngeal cancer.

## Conclusion

Dual inhibition of EGFR and HER3 by duligotuzumab was not associated with improved efficacy compared to cetuximab, neither in all randomized nor biomarker positive patients. Our data suggest that HPV-negative SCCHN but not HPV-positive SCCHN may respond to cetuximab or duligotuzumab, though the addition of HER3 inhibition does not significantly improve response but may increase the frequency of select lower grade GI toxicities.

## Author Contributions

Conception and design: PH, AK, BM, EP, AP, DR, and JV. Collection and assembly of data: MA, DC, JF, AJ, BM, CN, PO, CO, EP, AP, DR, AU, and LW. All authors contributed to data analysis and interpretation. All authors contributed to manuscript writing. All authors provided final approval of manuscript and its content.

## Conflict of Interest Statement

JF had leadership at Glycotope GmbH; LW had consultant roles at Ashion, Eisai, and Loxo; CO received research funding from Genentech, Inc., and participated as PI in different clinical studies; AU received honoraria from BMS, Astrazeneca, Amgen, and Teva, had consulting or advisory role at Teva and BMS, and received travel and/or accommodation expenses from Astellas and Teva; AJ has no disclosures; DR received research funding from Genentech, Merck, and Threshold; CN has no disclosures; PH and the University of Wisconsin received laboratory research support from Genentech, Inc.; TC has no disclosures; DC has no disclosures; PO has no disclosures; WH, AK, MA, EP, BM, and AP are employees of Genentech, Inc., South San Francisco, CA, USA and stockholders of Roche; JV participated in Advisory Boards at AstraZeneca, Boehringer-Ingelheim, Debiopharma, Merck-Serono, PCI Biotech, Merck Sharp & Dome Corp., Genentech, Pierre Fabre, Vaccinogen, Innate Pharma, Synthon Biopharmaceuticals, F-Star Biotechnology Ltd., and SMS-Oncology and received lecturer fees from Merck-Serono and Vaccinogen.
